# Pathogenic characteristics of sputum and bronchoalveolar lavage fluid samples from patients with lower respiratory tract infection in a large teaching hospital in China: a retrospective study

**DOI:** 10.1186/s12890-020-01275-8

**Published:** 2020-08-31

**Authors:** Zheng Peng, Jin’an Zhou, Lei Tian

**Affiliations:** 1 Department of Clinical Laboratory, Tongji Hospital, Tongji Medical College, Huazhong University of Science and Technology, Wuhan, Hubei Province China; 2 Department of Blood Transfusion, Tongji Hospital, Tongji Medical College, Huazhong University of Science and Technology, Wuhan, Hubei Province China

**Keywords:** Lower respiratory tract infection, Bronchoalveolar lavage fluid, Sputum

## Abstract

**Background:**

Lower respiratory tract infection (LRIs) is very common both in terms of community-acquired infection and hospital-acquired infection. Sputum and bronchoalveolar lavage fluid (BALF) are the most important specimens obtained from patients with LRI. The choice of antibiotic with which to treat LRI usually depends on the antimicrobial sensitivity of bacteria isolated from sputum and BALF. However, differences in the antimicrobial sensitivity of pathogens isolated from sputum and BALF have not been evaluated.

**Methods:**

A retrospective study was conducted to analyze the differences between sputum and BALF samples in terms of pathogen isolation and antimicrobial sensitivity in hospitalized patients with LRI.

**Results:**

Between 2013 and 2015, quality evaluation of sputum samples was not conducted before performing sputum culture; however, between 2016 and 2018, quality evaluation of sputum samples was conducted first, and only quality-assured samples were cultured. The numbers of sputum and BALF in 2013–2015 were 15,549 and 1671, while those in 2016–2018 were 12,055 and 3735, respectively. The results of pathogen culture showed that *Pseudomonas aeruginosa, Acinetobacter baumannii, Klebsiella pneumoniae, Staphylococcus aureus, Hemophilus influenzae, Escherichia coli, Stenotrophomonas maltophilia*, and *Streptococcus pneumoniae* were in the top ten pathogens isolated from sputum and BALF. An antimicrobial susceptibility test showed that the susceptibility of BALF isolates to most antibiotics was higher compared with the susceptibility of sputum isolates, especially after quality control of sputum samples (2016–2018).

**Conclusions:**

Our findings suggest that caution is needed in making therapeutic choices for patients with LRI when using antimicrobial sensitivity results from sputum isolates as opposed to BALF isolates.

## Background

Lower respiratory tract infection (LRI) is the most common infectious disease of the respiratory tract [1, 2]. Irrational use of antibiotics delays infection and can even lead to bacterial resistance [1, 2]. According to data from China Antimicrobial Resistance Surveillance System in 2015, the major specimen obtained from inpatients who attended respiratory departments in China was sputum (81.6%, 41,131/50,417) [1, 2]. Owing to the convenience of specimen collection, sputum has always been the most common specimen obtained in clinical microbiology laboratories in China. Sputum specimens are easily affected by oral colonization flora; thus, it is difficult to judge whether sputum culture isolates are indicative of infection, colonization or contamination. Because of this, it is difficult for clinicians and laboratory physicians to evaluate the significance of sputum culture results.

Bronchoalveolar lavage fluid (BALF) cultures are a reliable method to determine the bacterial etiology of LRI [3]. Whether there is a difference between the culture results obtained from sputum samples and BALF is currently unclear.

At our hospital, no quality evaluation of sputum samples was performed prior to 2015. However, since 2016, we have carried out quality evaluation of sputum samples. Only quality-assured samples can be used for sputum culture. Now, it is well known that unqualified sputum specimens, such as saliva, have no significance on bacterial culture. The industry has reached a consensus that sputum cultures without microscopic examination are of no value [4]. The qualified interpretation of sputum specimens in this study was based on Chinese standards [5].

Since 2016, smear microscopy has been used to examine each sputum specimen for culture. Sputum specimens satisfying the following three conditions are treated as qualified specimens: 1) specimen with ≥25 white blood cells (WBC) per average low-power field (LPF) and < 25 squamous epithelial cells (EPI) per LPF; 2) a ratio of WBC to EPI of > 10:1 and a predominance of single-form bacteria; 3) EPI < 10 per LPF and presence of alveolar macrophages and columnar epithelial cells. In addition to these criteria, when EPI is > 10 per LPF, sputum specimens are considered to be unqualified.

Herein, we examined the differences in sputum- versus BALF-based bacterial isolation and antimicrobial susceptibility results among hospitalized patients in a large tertiary hospital in China between 2013 and 2018. This approach enabled us to provide a more rigorous evaluation of the results of sputum culture.

## Methods

### Study setting

Sputum culture and BALF culture results from 2013 to 2015 and 2016 to 2018 were analyzed retrospectively at our hospital. The most common pathogens isolated from sputum and BALF in 2013–2015 and 2016–2018 were compared. The sensitivities of the same pathogens from sputum and BALF to commonly used antibiotics were compared. All specimens were taken from the clinical departments of Tongji Hospital and sent to the Department of Laboratory Medicine. Isolation and antimicrobial sensitivity tests were carried out on pathogenic bacteria at the clinical microbiology laboratory in accordance with standardized protocols. To eliminate the influence of antibiotics on culture results, in principle, samples should be collected before the use of antibiotics.

### Data collection

For isolates from the same source in the same patient, only the first isolate was included in the analysis in accordance with Clinical and Laboratory Standards Institute (CLSI) M39 [6]. Strain identification was carried out via biochemical experiments, an automatic identification system (VITEK® 2 Compact, bioMérieux, Marcy-l’Étoile, France), and/or IVD-MALDI Biotyper® (Bruker, Karlsruhe, Germany) [7–9]. An antimicrobial susceptibility test was carried out and explained in accordance with CLSI 2018 using the disk diffusion method and the E test method [10]. ATCC 25922, 25923, 27853, 49247, 49619, 90028, 35218, 700603, and 29213 were used for quality control of indoor antimicrobial sensitivity tests, which were performed weekly. In accordance with CLSI M39, the antimicrobial sensitivity results of different antimicrobial agents were expressed as the sensitivity rate [6].

### Statistical analysis

All patient and strain information were stored using WHONET software. WHONET 5.6 software was used to analyze antimicrobial susceptibility data. SPSS 19.0 software was used to compare the susceptibility rate between BALF and sputum isolates. The Chi-squared test was used to compare the sensitivity rate. A *P* value of < 0.05 was considered statistically significant.

### Ethics approval and consent to participate

The study protocol was approved by the Tongji Hospital ethics committee for research in health. The Tongji Hospital ethics committee also waived the requirement for informed consent from patients due to the retrospective design of the study. All patient data were anonymized prior to analysis.

## Results

### Study population

The study population included more adults than children and more male patients than female ones. The composition of our study population likely reflected the characteristics of the average population with LRI seeking care in the context. As such, because smoking was associated with a higher risk of developing LRI and adult male individuals were more likely to be smokers, these subjects represented the largest proportion of patients included in the study.

### Etiological distribution

**Fig. 1:**
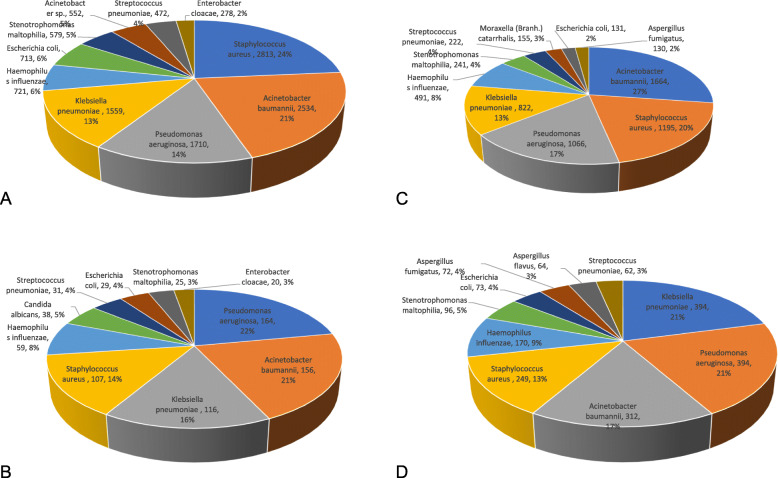
Distribution of pathogens isolated from sputum and BALF (top 10). **a** Distribution of pathogens from sputum specimens in 2013–2015. **b** Distribution of pathogens from BALF specimens in 2013–2015. **c** Distribution of pathogens from sputum specimens in 2016–2018. **d** Distribution of pathogens from BALF specimens in 2016–2018

The number of bacteria isolated from sputum and BALF in 2013–2015 was 12,957 and 848, respectively, compared with 6740 and 2239, respectively, in 2016–2018. In 2013–2015 and 2016–2018, *Pseudomonas aeruginosa, Acinetobacter baumannii, Klebsiella pneumoniae, Staphylococcus aureus, Hemophilus influenzae, Escherichia coli, Stenotrophomonas maltophilia*, and *Streptococcus pneumoniae* were in the top ten pathogens isolated from sputum and BALF (Fig. 1).

### Antimicrobial susceptibility

The susceptibility rates of *P. aeruginosa* in sputum and BALF to commonly used antibiotics were compared in 2013–2015 and 2016–2018. In 2013–2015, the sensitivity rates of *P. aeruginosa* in BALF to commonly used antibiotics were higher compared with sputum isolates, with the exception of ciprofloxacin and levofloxacin. In 2016–2018, with the exception of amikacin, gentamicin, and tobramycin, the sensitivity rates of BALF isolates to commonly used antibiotics were higher compared with the sensitivity rates of sputum isolates (Table 1).

**Table 1:** Sensitivity rate of *Pseudomonas aeruginosa* isolated from sputum and BALF to commonly used antibiotics

Antibiotics	2013–2015			2016–2018		
	BALF	Sputum	*P*	BALF	sputum	*P*
Piperacillin	75	57.3	< 0.01	68.2	55.8	*P* < 0.01
Cefoperazone/sulbactam	72.7	58.1	< 0.01	71.8	54.6	*P* < 0.01
Ticarcillin/clavulanic acid	40.3	12.4	< 0.01	40.3	10.8	*P* < 0.01
Piperacillin/tazobactam	79.3	62.8	< 0.01	74.6	61.7	*P* < 0.01
Cefoperazone	64.2	49.6	< 0.01	65.1	48.2	*P* < 0.01
Ceftazidime	80.5	64.5	< 0.01	73.4	62.3	*P* < 0.01
Cefepime	81.1	64.5	< 0.01	78.1	68.5	*P* < 0.01
Aztreonam	71.3	53.8	< 0.01	63.5	46.8	*P* < 0.01
Imipenem	72	63.2	0.01 < *P* < 0.05	68.6	57.4	*P* < 0.01
Meropenem	81	64.1	< 0.01	74.4	61.7	*P* < 0.01
Amikacin	88.4	72.7	< 0.01	87.6	86.4	*P* > 0.05
Gentamicin	82.1	61.8	< 0.01	80.5	79.4	*P* > 0.05
Tobramycin	88.4	70.4	< 0.01	87.8	84	*P* > 0.05
Ciprofloxacin	71.3	65	*P* > 0.05	75.6	67.1	*P* < 0.01
Levofloxacin	64.2	58.1	*P* > 0.05	68.9	61.2	*P* < 0.01
Trimethoprim sulfamethoxazole	21.6	7.8	< 0.01	14.3	5.5	*P* < 0.01
Minocycline	35	13.2	< 0.01	31.6	14	*P* < 0.01

The susceptibility of *A. baumannii* to commonly used antibiotics showed that isolates from BALF in 2013–2015 were more sensitive compared with sputum isolates in response to most antibiotics, with the exception of cefoperazone/sulbactam, ampicillin/sulbactam, imipenem, meropenem, gentamicin, ciprofloxacin, minocycline, and aztreonam. However, data from 2016 to 2018 showed that the susceptibility rates of BALF isolates to antibiotics, with the exception of piperacillin, ceftazidime, aztreonam, minocycline, and tigecycline, were higher compared with sputum isolates (Table 2).

**Table 2:** Sensitivity rate of Acinetobacter baumannii isolated from sputum and BALF to commonly used antibiotics

Antibiotics	2013–2015			2016–2018		
	BALF	sputum	*P*	BALF	sputum	*P*
Piperacillin	25.8	7.9	*P* < 0.01	2.2	2.3	*P* > 0.05
Cefoperazone/sulbactam	14.1	17.8	*P* > 0.05	9.3	6.1	0.01 < *P* < 0.05
Ampicillin/sulbactam	14.2	16.7	*P* > 0.05	9.3	5.7	0.01 < *P* < 0.05
Piperacillin/tazobactam	32.1	14.8	*P* < 0.01	7.1	4	0.01 < *P* < 0.05
Ceftazidime	27.1	8.4	*P* < 0.01	4.8	3.1	*P* > 0.05
Cefepime	32.1	16.6	*P* < 0.01	9	5.2	*P* < 0.01
Aztreonam	0.6	1.5	*P* > 0.05	0.6	0.2	*P* > 0.05
Imipenem	11	16.9	*P* > 0.05	9.6	5.5	*P* < 0.01
Meropenem	10.9	15.8	*P* > 005	9.3	5	*P* < 0.01
Amikacin	12.8	21.4	0.01 < *P* < 0.05	14.7	10	0.01 < *P* < 0.05
Gentamicin	9.6	14.8	*P* > 0.05	9.6	5.5	*P* < 0.01
Tobramycin	10.9	18.1	0.01 < *P* < 0.05	12.8	7.5	*P* < 0.01
Ciprofloxacin	15.4	17.4	*P* > 0.05	8.3	4.7	*P* < 0.01
Levofloxacin	31.4	18.2	*P* < 0.01	9	5	*P* < 0.01
Trimethoprim sulfamethoxazole	33.3	16.7	*P* < 0.01	10.3	6.7	0.01 < *P* < 0.05
Minocycline	38.7	33.8	*P* > 0.05	33.4	35.3	*P* > 0.05
Tegacycline	34.8	46.3	*P* < 0.01	43.4	46.4	*P* > 0.05

The sensitivity of *K. pneumoniae* to commonly used antibiotics was higher in BALF isolates compared with sputum isolates, with the exception of piperacillin, amikacin, gentamicin, tobramycin, ciprofloxacin, levofloxacin, and tigecycline in 2013–2015. In 2016–2018, with the exception of tigecycline, BALF isolates were more sensitive to commonly used antibiotics compared with sputum isolates (Table 3).

**Table 3:** Sensitivity rate of *Klebsiella pneumoniae* isolated from sputum and BALF to commonly used antibiotics

Antibiotics	2013–2015			2016–2018		
	BALF	Sputum	*P*	BALF	Sputum	*P*
Piperacillin	31	23.7	*P* > 0.05	22.6	7	*P* < 0.01
Amoxicillin/clavulanic acid	59.6	43	*P* < 0.01	53.8	21.3	*P* < 0.01
Cefoperazone/sulbactam	70.4	53.9	*P* < 0.01	56.1	24.1	*P* < 0.01
Ampicillin/sulbactam	53.4	33.8	*P* < 0.01	49.7	15.7	*P* < 0.01
Piperacillin/tazobactam	75	61.1	*P* < 0.01	58.5	28.6	*P* < 0.01
Cefazolin	36.2	25.1	*P* < 0.01	42	12.8	*P* < 0.01
Cefuroxime	56	34	*P* < 0.01	49.9	16.6	*P* < 0.01
Ceftazidime	69.8	50.4	*P* < 0.01	54.8	23.8	*P* < 0.01
Cefotaxime	57.4	36.2	*P* < 0.01	51.9	17.9	*P* < 0.01
Cefepime	61.4	38.2	*P* < 0.01	53.8	19.2	*P* < 0.01
Cefoxitin	77.6	65.6	*P* < 0.01	59.2	32.6	*P* < 0.01
Aztreonam	69	48.9	*P* < 0.01	54.8	21.4	*P* < 0.01
Imipenem	90.5	81.3	0.01 < *P* < 0.05	67.3	40.3	*P* < 0.01
Meropenem	90.5	81	0.01 < *P* < 0.05	67.7	40.6	*P* < 0.01
Amikacin	87.8	83.3	*P* > 0.05	70.6	52.2	*P* < 0.01
Gentamicin	66.4	58.4	*P* > 0.05	61.4	38.5	*P* < 0.01
Tobramycin	61.2	55.5	*P* > 0.05	57.8	36.6	*P* < 0.01
Ciprofloxacin	69.6	60.4	*P* = 0.05	55.3	22.6	*P* < 0.01
Levofloxacin	75.9	71.2	*P* > 0.05	59.9	28.3	*P* < 0.01
Trimethoprim sulfamethoxazole	67.2	56.9	0.01 < *P* < 0.05	64.5	55.7	*P* < 0.01
Tegacycline	87.2	87.1	*P* > 0.05	92.3	89.2	*P* > 0.05

From 2013 to 2015, the sensitivity of *S. aureus* isolated from BALF was higher in response to some antibiotics compared with the sensitivity of *S. aureus* isolated from sputum isolates, with the exception of penicillin, gentamicin, levofloxacin, fosfomycin, erythromycin, clindamycin, and tigecycline. However, in 2016–2018, bacteria isolated from BALF isolates were more sensitive than bacteria isolated from sputum isolates to common antibiotics, with the exception of trimethoprim/sulfanilamide and tigecycline (Table 4).

**Table 4:** Sensitivity rate of *Staphylococcus aureus* isolated from sputum and BALF to commonly used antibiotics

Antibiotics	2013–2015			2016–2018		
	BALF	sputum	*P*	BALF	sputum	*P*
Penicillin G	1.9	1.5	*P* > 0.05	3.2	0.9	*P* < 0.01
Oxacillin	30.2	19.9	*P* < 0.01	32.5	15.6	*P* < 0.01
Ampicillin/sulbactam	30.8	19.4	*P* < 0.01	31.6	15.6	*P* < 0.01
Cefazolin	30.8	19.3	*P* < 0.01	30.9	15.5	*P* < 0.01
Cefuroxime	30.8	19.9	*P* < 0.01	32.5	15.6	*P* < 0.01
Cefoxitin	30.8	20	*P* < 0.01	32.5	15.5	*P* < 0.01
Gentamicin	32.7	24.6	*P* > 0.05	41.6	24	*P* < 0.01
Tobramycin	32.7	23.1	0.01 < *P* < 0.05	39.7	22.6	*P* < 0.01
Rifampicin	47.7	30.3	*P* < 0.01	88.7	83.3	0.01 < *P* < 0.05
Levofloxacin	28.3	23.8	*P* > 0.05	39.8	19.1	*P* < 0.01
Trimethoprim sulfamethoxazole	92.5	96.9	0.01 < *P* < 0.05	97.2	96.9	*P* > 0.05
Fosfomycin	83.5	80.6	*P* > 0.05	51.9	37.7	*P* < 0.01
Clindamycin	68.9	69.6	*P* > 0.05	35.7	24.1	*P* < 0.01
Erythromycin	46.7	52.6	*P* > 0.05	26.5	15.6	*P* < 0.01
Linezolid	100	100		100	100	
Vancomycin	100	100		100	100	
Teicoplanin	100	100		100	100	
Tegacycline	92.9	90.2	*P* > 0.05	94.6	93.6	*P* > 0.05

## Discussion

Our study found that the main pathogens isolated from sputum samples and BALF were roughly the same. However, the sensitivities of the main pathogens to commonly used antibiotics were different. The sensitivity of BALF isolates to the most commonly used antibiotics was higher compared with the sensitivity of sputum isolates, especially after quality control of sputum samples.

This study found that the main pathogens isolated from sputum and BALF were *P. aeruginosa, A. baumannii, K. pneumoniae, S. aureus, H. influenzae, E. coli, S. maltophilia*, and *S. pneumoniae*. The high isolation rate of *A. baumannii* may be related to the fact that most patients were hospitalized. *A. baumannii* is the main pathogen associated with hospital-acquired infection [11]. On the contrary, *S. pneumoniae* is the main pathogen associated with community-acquired infection and pediatric infection [12]. The study population mainly comprised adult patients, and the number of hospitalized patients was far greater than the number of outpatients, which may be related to the low isolation rate of *S. pneumoniae*.

The results of antimicrobial sensitivity tests showed that the sensitivity of BALF isolates to commonly used antibiotics was higher compared with the sensitivity of sputum isolates. Comparing the data of samples obtained in 2016–2018 with those of samples obtained in 2013–2015, this trend was more obvious in 2016–2018. The differences in sensitivity between BALF and sputum isolates may be related to the presence of respiratory colonization in sputum samples. We also found that, even if sputum samples were qualified, the sensitivity of sputum isolates to commonly used antibiotics was still significantly lower compared with the sensitivity of BALF isolates. This finding suggests that caution should be exercised when evaluating the results of sputum culture, especially when selecting antibiotics on the basis of the susceptibility of pathogens found in sputum cultures.

The clinical significance of sputum culture has always been controversial, making therapeutic decision-making even more challenging. Owing to contamination with oral flora, it is difficult to judge on whether pathogenic bacterial isolates are indicative of infection or colonization.

In this study, we evaluated the significance of sputum culture from a new perspective. This study compared the differences in pathogens and antimicrobial sensitivity between sputum samples and BALF, and compared the differences before and after quality control of sputum samples. Studies have shown that the results of culture and antimicrobial sensitivity are quite different between qualified sputum specimens and BALF. BALF was obtained via fiberoptic bronchoscopy and could represent the status of LRI infection. However, sputum specimens are easily contaminated by colonies in the upper respiratory tract. Therefore, clinicians need to be very careful when diagnosing and treating LRI on the basis of the results of sputum culture.

Sputum specimens are not optimal specimens from the viewpoint of LRI diagnosis. Doctors obtain biopsy specimens via fiberoptic bronchoscopy, the results of which could identify LRI. However, fiberoptic bronchoscopy is invasive and is not suitable for every patient. At present, qualified sputum specimens together with some invasive surgical techniques (e.g., transtracheal aspiration, bronchoalveolar lavage, protected brush samples) are acceptable in the global LRI surveillance project [13]. In the face of LRI, which is the most appropriate type of specimen is a question worth considering. The American Association of Pediatric Infectious Diseases specifies that blood cultures should be tested for moderate-to-severe community-acquired pneumonia in children, especially those with complex pneumonia [14]. However, at present, the rate of blood culture in Chinese patients with LRI is low. A multicenter study carried out in China showed that blood culture isolates accounted for only 5.3% of all specimen types [1, 2]. For LRIs, the question of whether one should perform blood culture or sputum culture is something every clinician often considers. Different strategies should be adopted for different patients. Outpatient blood culture and sputum culture are not routinely required. For inpatients with low-severity LRI, only sputum culture is needed. For inpatients with moderate severity LRI and no intensive care unit sputum culture, blood culture, legionella urinary antigen, and pneumococcal urinary antigen should be examined routinely. For inpatients in the intensive care unit with high-severity LRI, invasive sampling should also be performed in addition to all the above mentioned tests [15].

There are several limitations to the present study that should be highlighted. First, it was difficult to evaluate colonization of sputum culture. We were not able to determine whether the isolated strains of sputum samples were colonized bacteria or infectious pathogens. Second, in this study, no distinction was made among natural expectoration, induced sputum, or sputum aspiration. We hope that, in future, we can use more rigorous methods to evaluate sputum samples.

## Conclusions

Although the main pathogens isolated from sputum and BALF were the same, their antimicrobial sensitivities were different, even for qualified sputum specimens. Depending on the results of sputum culture, caution should be exercised when deciding on the most appropriate treatment options for patients.

## Data Availability

The datasets used and/or analyzed during the current study are available from the corresponding author on reasonable request.

## Abbreviations

LRIs: Lower respiratory tract infections
CARSS: China Antimicrobial Resistance Surveillance System
WBC: White blood cells
LPF: Low-power field
EPI: Squamous epithelial cells
BALF: Bronchoscopic lavage fluid
CLSI: Clinical and Laboratory Standards Institute
